# Full-space Cloud of Random Points with a Scrambling Metasurface

**DOI:** 10.1038/s41377-018-0064-3

**Published:** 2018-09-12

**Authors:** Zile Li, Qi Dai, Muhammad Q. Mehmood, Guangwei Hu, Boris Luk’ yanchuk, Jin Tao, Chenglong Hao, Inki Kim, Heonyeong Jeong, Guoxing Zheng, Shaohua Yu, Andrea Alù, Junsuk Rho, Cheng-Wei Qiu

**Affiliations:** 1 School of Electronic Information, Wuhan University, Wuhan, 430072 China; 2 NOEIC, State Key Laboratory of Optical Communication Technologies and Networks, Wuhan Research Institute of Posts and Telecommunications, Wuhan, 430074 China; 3 Department of Electrical Engineering, Information Technology University of the Punjab, Ferozpur Road, 54000 Lahore, Pakistan; 4 Department of Electrical and Computer Engineering, National University of Singapore, 4 Engineering Drive 3, Singapore, 117583 Singapore; 5 Advanced Science Research Center, City University of New York, New York, 10031 USA; 6 Data Storage Institute, A*STAR (Agency for Science, Technology and Research), 2 Fusionopolis Way, #08-01, Innovis, 138634 Singapore; 7 Department of Mechanical Engineering, Pohang University of Science and Technology (POSTECH), Pohang, 37673 Republic of Korea; 8 Department of Chemical Engineering, Pohang University of Science and Technology (POSTECH), Pohang, 37673 Republic of Korea; 9 National Institute of Nanomaterials Technology (NINT), Pohang, 37673 Republic of Korea

## Abstract

With the rapid progress in computer science, including artificial intelligence, big data and cloud computing, full-space spot generation can be pivotal to many practical applications, such as facial recognition, motion detection, augmented reality, etc. These opportunities may be achieved by using diffractive optical elements (DOEs) or light detection and ranging (LIDAR). However, DOEs suffer from intrinsic limitations, such as demanding depth-controlled fabrication techniques, large thicknesses (more than the wavelength), Lambertian operation only in half space, etc. LIDAR nevertheless relies on complex and bulky scanning systems, which hinders the miniaturization of the spot generator. Here, inspired by a Lambertian scatterer, we report a Hermitian-conjugate metasurface scrambling the incident light to a cloud of random points in full space with compressed information density, functioning in both transmission and reflection spaces. Over 4044 random spots are experimentally observed in the entire space, covering angles at nearly 90°. Our scrambling metasurface is made of amorphous silicon with a uniform subwavelength height, a nearly continuous phase coverage, a lightweight, flexible design, and low-heat dissipation. Thus, it may be mass produced by and integrated into existing semiconductor foundry designs. Our work opens important directions for emerging 3D recognition sensors, such as motion sensing, facial recognition, and other applications.

## Metasurfaces: scrambling light for 3D detection and recognition

Firing light at a manufactured "metasurface"—one carrying patterns at a smaller scale than the wavelength of the light—fills large volumes of space with defined points of light, potentially improving 3-D recognition and sensor applications. Cheng-Wei Qui and colleagues at the National University of Singapore, with co-workers across Asia and in the USA, created their unique metasurface from amorphous silicon. Light is scattered from and transmitted through the material to generate a cloud of data points in the surrounding space in which the structure and motion of objects under study can be analyzed. The initial development work with this “scrambling metasurface” suggests it could improve pattern recognition, including face recognition, motion detection and augmented reality applications. The researchers describe how their innovation overcomes significant limitations of existing methods in these fields.

## Introduction

The last decade has witnessed the emergence of novel technologies in computer science, ranging from big data, cloud computation, and machine learning to artificial intelligence. These require increasingly powerful electronic or photonic devices to enable important applications, such as augmented reality, point-to-point medical electronics, and wearable devices. In all these applications, there is a strong need to create or collect data all over space, i.e., to enable point clouds. A point cloud refers to a set of data points in space, usually in three dimensions, based on which a growing number of important applications have recently been achieved, such as motion detection and facial recognition using commercially available products such as Kinect (Microsoft Corp.)^1^ and iPhone X (Apple Corp.). Common methods to generate a point cloud or, equivalently, spot arrays in optics use diffractive optical elements (DOEs)^2,3^ or light detection and ranging (LIDAR)^4,5^, on which the two aforementioned commercial products are based. The different depths etched on DOEs can create a phase profile, enabling the diffracted beam to form spot arrays on one side, either the transmission or reflection side, similar to a Lambertian surface^6^. Once the scattered beams are captured by the sensors, the computer program can search the encoded information and determine the morphology of the detected objects. However, this approach leads to several disadvantages, such as fabrication difficulty, the inherent limitation of spots spanning only a 2π space, and fundamental limits on the thickness of these devices (at least comparable to the wavelength), which may seriously hinder various applications. Even though the point cloud generated by LIDAR can cover the full space by scanning the azimuth and elevation, the complex and bulky scanning system obstructs the miniaturization of the point cloud generator, which is critical for light portable devices. Thus, to further improve and commercialize novel technologies based on point clouds, apart from the software, new devices must be developed. These should meet demanding requirements: compatibility for integration into traditional silicon-based semiconductor devices, ultrathin form factors to ensure compactness and miniaturization for future trends in light portable devices, commercially viable fabrication and, most importantly, functionality to generate millions of data points to increase the information capacity in full space.

Metasurfaces, generally considered the two-dimensional counterpart of bulk metamaterials, have recently been exploited to manipulate the properties of light, especially for phase engineering. Recent work has demonstrated that by exploiting the Pancharatnam–Berry (PB) phase introduced by local rotations of the effective half-wave plate residing in the unit cell, one can achieve high-numerical-aperture metalens^7–11^, high-efficient meta-holograms^12–22^ and beam steering^23,24^, or polarization-independent diffusion and switched functionality^25–27^. There are also some reported works related to metasurfaces for Dammann arrays^28,29^ and selective diffraction order generation^30^ to form uniform or nonuniform spot arrays. However, no work has demonstrated that the diffracted beam can be extended to occupy the full space, which is fundamentally imperative in point cloud generation.

**Fig. 1:**
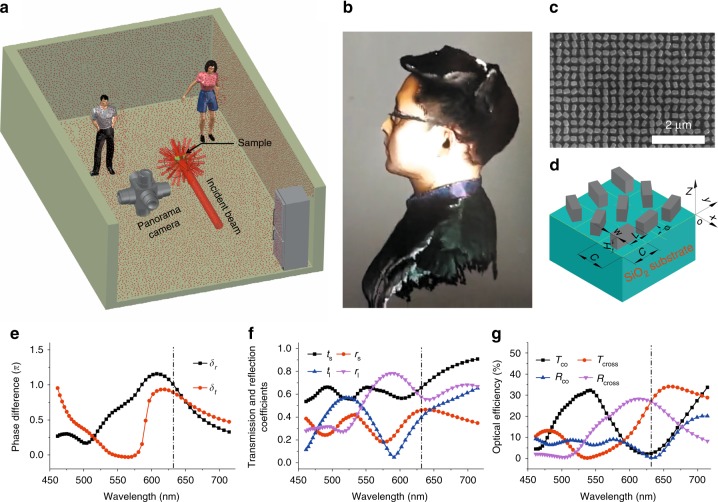
Generation of full-space cloud of random points for 3D sensing applications. **a** A typical motion sensing application scene and a scrambling metasurface used to generate random spot arrays filling the 4π spherical space. **b** Application of random point cloud-based 3D application for facial recognition. **c** SEM photo of the scrambling metasurface sample (partial view). **d** Illustration of a full-space scrambling metasurface with amorphous silicon nanobrick arrays sitting on a fused silica substrate. All nanobricks are periodically arranged and have the same dimensions with height *H* = 277 nm, length *L* = 230 nm, width *W* = 124 nm, and cell size *C* = 300 nm, but with different orientation angles. **e** Simulated phase differences of the output beams between the long and short axes for both reflection (*δ*_r_) and transmission (*δ*_t_). **f** Simulated reflection and transmission coefficients versus wavelength when an incident beam is linearly polarized along the long and short axes of the nanobrick. **g** Simulated efficiency of the co-polarized (*R*_co_) and cross-polarized (*R*_cross_) response in reflection and the co-polarized (*T*_co_) and cross-polarized (*T*_cross_) response in transmission versus wavelength

Lambertian surfaces, which appear as uniformly bright from all directions and reflect the entire incident light, can only operate in half space and as a half-space reflective spot generator. Inspired by the response of Lambertian surfaces, we proposed the realization of a scrambling metasurface to support a cloud of random points in full space with a distribution analogous to Lambertian scattering with compressed information density. This design overcomes the aforementioned limitations of DOEs and LIDAR. Owing to the judicious pattern design to achieve rotationally symmetric spot arrays in the far field, our scrambling metasurface can simultaneously operate on the transmission and reflection sides regardless of the polarization of incident light, serving as a full-space metasurface. As a proof-of-concept, more than 4044 random spots can be experimentally observed in the whole space, covering angles at nearly 90°. Our scrambling metasurface is made of amorphous silicon with a height smaller than half the wavelength, which may be mass produced by mature nanofabrication techniques, such as photolithography. We believe that our work can provide ultrathin, low-dissipation, silicon-compatible, light and efficient optical devices for point cloud generation. When combined with computer algorithms for positioning the cloud of random points, this work may find important applications in emerging technologies, such as motion sensing and facial recognition, which are illustrated in Fig. 1a, b, respectively.

## Results

### Design of scrambling metasurfaces

Conventional optical devices work in either transmission mode or reflective mode, but not both. To extend the diffraction response of geometric metasurfaces, electromagnetic resonances sustained by dielectric nanobrick metasurfaces are employed to control the reflection of light. Figure 1d shows a schematic diagram of a scrambling metasurface that consists of a two-layer structure: a ground substrate and a top layer of nanobricks. To understand the working principle of such a scrambling metasurface, we use Jones calculus (a nanobrick can be treated as a birefringent element) to express the reflection and transmission as

1 $${\mathrm{G}}_r = \left[ {\begin{array}{*{20}{c}} {r_l} & 0 \\ 0 & {r_se^{i\delta _r}} \end{array}} \right],{\mathrm{G}}_t = \left[ {\begin{array}{*{20}{c}} {t_l} & 0 \\ 0 & {t_se^{i\delta _t}} \end{array}} \right]$$

where *r*_s_, *t*_s_, *r*_l_, and *t*_l_ are the reflection and transmission coefficients when the waves propagate with polarization along the short (*s*) and long (*l*) axes of the nanobricks, and *δ*_r_ and *δ*_t_ are the phase differences between two orthogonal directions of the reflected and transmitted light, respectively.

When a circularly polarized (CP) incident beam, say left-handed CP light (LCP), shines on the metasurface, the output beam is divided into four parts: two sub-beams with the same handedness as the incident beam that experience no additional phase delay for both reflection and transmission, called co-polarized beams, and two with opposite handedness, i.e., right-handed CP light (RCP), which carry an additional phase delay of 2*ϕ* for both reflection and transmission, where *ϕ* is the orientation angle of the nanobrick, called cross-polarized beams. Thereby, the reflected and transmitted wave can be mathematically described in a matrix form under the CP basis:

2a $$\left[ {\begin{array}{*{20}{c}} {E_r^{LCP}} \\ {E_r^{RCP}} \end{array}} \right] = \left[ {\begin{array}{*{20}{c}} {R_{co}} & {R_{cross}e^{ - i2\phi }} \\ {R_{cross}e^{ - i2\phi }} & {R_{co}} \end{array}} \right]\left[ {\begin{array}{*{20}{c}} {E_{inc}^{LCP}} \\ {E_{inc}^{RCP}} \end{array}} \right]$$

2b $$\left[ {\begin{array}{*{20}{c}} {E_t^{LCP}} \\ {E_t^{RCP}} \end{array}} \right] = \left[ {\begin{array}{*{20}{c}} {T_{co}} & {T_{cross}e^{ - i2\phi }} \\ {T_{cross}e^{ - i2\phi }} & {T_{co}} \end{array}} \right]\left[ {\begin{array}{*{20}{c}} {E_{inc}^{LCP}} \\ {E_{inc}^{RCP}} \end{array}} \right]$$

Here, (*R*_coss_, *T*_cross_) and (*R*_co_, *T*_co_) are the polarization conversion efficiency of the output beams’ (both reflected and transmitted waves) cross-polarization component with phase delay and co-polarization component without phase delay, respectively. The polarization conversion efficiency can be deduced as:

3 $$\left\{ {\begin{array}{*{20}{c}} {R_{cross} = |\frac{{r_l - r_se^{i\delta _r}}}{2}|^2} \\ {R_{co} = |\frac{{r_l + r_se^{i\delta _r}}}{2}|^2} \end{array}} \right.{\it{,}}\left\{ {\begin{array}{*{20}{c}} {T_{cross} = |\frac{{t_l - t_se^{i\delta _t}}}{2}|^2} \\ {T_{co} = |\frac{{t_l + t_se^{i\delta _t}}}{2}|^2} \end{array}} \right.$$

By judiciously designing the structural parameters of the nanobrick, the co-polarized parts can be suppressed, and the ratio of the cross-polarized parts between the transmission and reflection can be controlled. In particular, if *T*_cross_ = *R*_cross_ and *T*_co_ = *R*_co_ = 0 or, equivalently, *δ*_r_ = *δ*_t_ = *π* and and *r*_l_ = *r*_s_ = *t*_l_ = *t*_s_, then the reflectivity equals the transmissivity, meaning that the reflection and transmission matrices in Eqs. 2a and 2b are identical, and all the incident CP light is converted into cross-polarized beams traveling equally in the two opposite directions normal to the metasurface plane. Our previous literature result^21^ has already shown that the above conditions are non-trivial, and the optimal polarization conversion efficiency with the designed phase control can be achieved while *δ*_r_ = *δ*_t_ = ***π*** and *r*_l_ = *r*_s_ = *t*_l_ = *t*_s_ are not strictly observed. Thus, we designed the unit cells with the geometric parameters shown in Fig. 1d, and the full wave simulation result (see Methods) is shown in Fig. 1e–f. As seen in Fig. 1e, the phase differences between the long and short axes approach π for both reflection and transmission. In addition, Fig. 1f shows the simulated reflection and transmission coefficients when a normally incident beam is polarized along the long and short axes of the nanobrick. Nevertheless, the polarization conversion efficiency of both the reflection and transmission reaches 27%, whereas the unwanted co-polarized beams contributing to the zero-th order diffraction can be suppressed to a negligible level below 3% (0.4% for reflection and 2.6% for transmission), as shown in Fig. 1g. Therefore, each nanobrick can convert a CP incident beam into the opposite handedness with a phase delay exactly twice the orientation angle of the nanobrick in both transmission and reflection^19,20^, while only a negligible residual component with the same handedness exist. By simply changing the orientation angle cell by cell, we can fully manipulate the beam and steer it into any desired direction. Meanwhile, it should be noted that for our metasurface, the reflection and transmission matrices are Hermitian conjugate with zeros in the diagonal component. Therefore, the diffracted patterns formed by LCP and RCP light will coincide with each other if the target pattern is designed with rotational symmetry (see Methods).

### Demonstration of a blazed grating and a 2 × 2 beam splitter

**Fig. 2:**
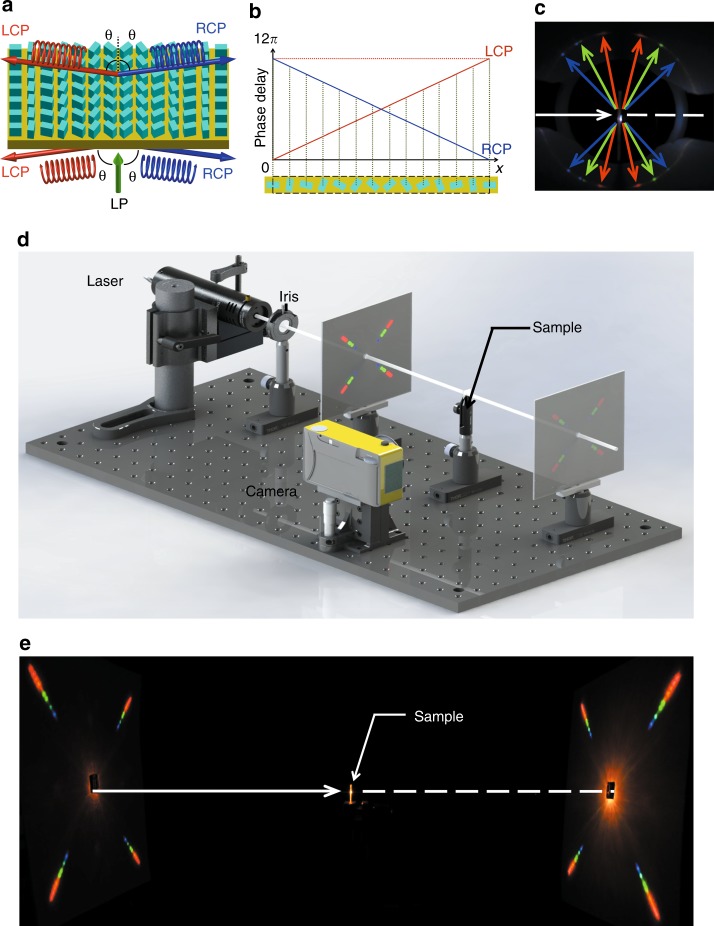
Schematic illustration and experimental results of the scrambling metasurface-based blazed grating and 2 × 2 beam splitter. **a** The working principle of the scrambling metasurface-based blazed grating consists of amorphous silicon nanobricks sitting on a fused silica substrate. The incident LP beam is diffracted into different directions depending on the handedness of the output sub-beams. **b** The phase delay of each nanobrick. In one period, the grating contains 13 nanobricks, spaced at 300 nm (center-to-center) and rotated by an angle step of 6π/13 cell by cell. **c** Experimental results of the transflective blazed grating illuminated by a supercontinuum laser with incident wavelengths of 470 nm, 570 nm, and 630 nm (design wavelength). The white arrows indicate the direction of incident light. **d** The experimental setup to characterize the scrambling metasurface-based beam splitter. A supercontinuum light source was used to illuminate the sample. **e** Experimental images with the supercontinuum illumination

A schematic illustration of a scrambling metasurface-based blazed grating is shown in Fig. 2a–c. As a linearly polarized (LP) beam can be treated as a combination of LCP and RCP beams with equal intensity, such a grating can diffract an incident beam into four sub-beams with symmetrical propagation directions owing to the spin-dependent nature of the PB phase and with equal intensity due to the equal polarization conversion efficiency, as shown in Fig. 2a. It is interesting to highlight that the incident light can be naturally polarized as well, as it can be decomposed into circular polarization components with equal weight, and that our metasurface can route them to the desired directions. The experimental setup and results for the blazed grating are shown in Fig. 2c. The diffraction angle with a designed order (*m* = 6) increases as the operation wavelength varies from 470 nm to 650 nm and then disappears above 650 nm since the diffraction angle exceeds 90° (see the video in Supplementary Movies and Supplementary Information). This result validates that the diffraction beams of our scrambling metasurfaces can radiate in the full space. More details about the design and performance are provided in the Supplementary Information.

By adding another phase gradient enabled by rotating the geometry perpendicular to the above grating direction, we can extend the functionality of the blazed grating to steer the incident beam into 2 × 2 spot arrays with uniform intensity in both transmission and reflection spaces, which could serve as proof-of-concept beam splitting for point cloud generation. With the structural parameters of a unit cell demonstrated in Fig. 1, we realized a 2 × 2 beam splitter using scrambling metasurfaces. The beam splitter was designed with a unit cell size of 300 × 300 nm^2^, a grating period of 1.5 × 1.5 μm^2^ and an operational wavelength of 630 nm. The experimental setup used to characterize the performance of the transflective beam splitter is shown in Fig. 2d. A normally incident beam from a supercontinuum light source (YSL SC-pro) illuminates the sample after passing through an iris. The reflective and transmissive sub-beams with different diffraction orders can be observed in the far field captured by the camera. Figure 2e shows the diffractive 2 × 2 spot arrays on screens at a distance of 150 mm from the sample both in reflective and transmissive spaces with wavelengths from 470 nm to 650 nm at a 20 nm interval. According to the grating equation, the sub-beams with the same diffraction orders but different wavelengths differ in diffraction angles. It can also be noted that the optical power distribution is almost uniform both in the reflective and transmissive spaces owing to the equal polarization conversion efficiency for both transmission and reflection.

### Demonstration of a cloud of random points in full space

To demonstrate the flexibility of our proposed beam manipulation technique, we designed a random point cloud (RPC) metasurface, based on which the diffraction beams are projected into a 4*π* solid angle. As a proof-of-concept, we took 4044 random spot arrays with uniform intensity as the target pattern. The density of the point cloud can be increased by extending the metasurface aperture as a function of the requirements of practical applications. Another remarkable result is that our design of the RPC with rotational symmetry can help eliminate the polarization dependence existing in the PB phase elements. More details are described in the Methods. To produce spot arrays and avoid laser speckle, the RPC should be designed in periodic structures. In this design, the RPC consists of 10 × 10 periods, each with dimensions of 30 × 30 μm^2^ (100 × 100 nanobrick arrays). As each cell consists of a sub-wavelength nanostructure (300 × 300 nm^2^) with the same height, the diffracted beam can fill the whole spherical space. The phase distribution design and conversion efficiency, signal-to-noise ratio and uniformity were taken as merit functions for optimization^20^. As the phase delay is determined solely by the orientation of the nanobrick, continuous phase levels were used to obtain a high performance of the RPC.

**Fig. 3:**
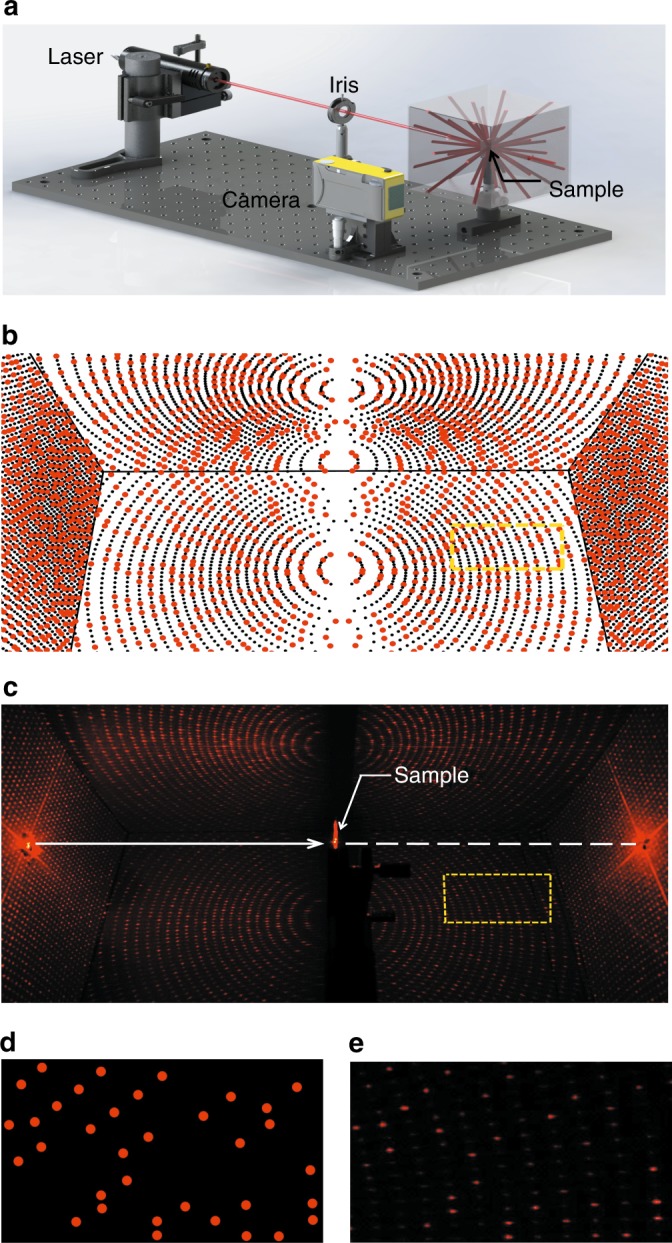
Experimental setup, simulation, and experimental results for an GCP in 4π space based on a full-space scrambling metasurface. **a** The experimental setup used to characterize the scrambling metasurface-based RPC. A linearly polarized laser beam with a wavelength of 632.8 nm passes through an iris and illuminates a sample of the RPC. The transflective diffraction sub-beams fill a square box with two holes for beam entrance and exit. **b**–**c** Simulated spot arrays filling a box, and experimentally obtained images captured by a "visible" camera. The red and black spots indicate the spatial position of the design and noise beam spots, respectively. The yellow box on the border marks the area of the zoomed-in view. **d**–**e** Simulated spot arrays and experimentally obtained images, with an enlarged zoom of a partial view in transmission space. The operation wavelength is 632.8 nm

The incident beam from a He-Ne laser illuminates the scrambling metasurface sample, which is placed in the center of a square box. Then, the diffracted sub-beams fill the whole 4*π* space according to our design because the diffraction angle reaches 90°, which is also demonstrated in the blazed grating (see Supplementary Information). In Fig. 3b–e, both the simulated and measured beam spots, including zoomed-in views of the spots in the transmissive space, show good agreement. This demonstrates the extremely high fidelity of the RPC generator based on the scrambling metasurface.

## Discussion

Diffraction efficiency is an important aspect in the performance of the scrambling metasurface. In our presented work, the diffraction efficiency, defined as the amount of power coupled to the desired diffraction orders divided by the incident beam power, was measured to be approximately 30% for the above 2 × 2 beam splitter (it is difficult to measure the efficiency of an RPC, as the diffracted sub-beams fill the whole space). Although this value may be lower than what is required for practical three-dimensional (3D) sensing applications, we must note that this issue is associated with fabrication challenges, rather than a fundamental limit. It is believed that by exploiting low-loss materials, such as monocrystalline silicon^31^ or TiO_2_^32^, the efficiency may be greatly increased. As an example to demonstrate the feasibility, we designed another scrambling metasurface in the infrared range (830 nm) that achieves 85% efficiency (shown in Supplementary Fig. S2). Thus, the scattered point information could be employed to determine the morphology of the structures and be applied to real-world applications.

In our work, the proposed scrambling metasurface can be treated as an accurate phase modulator and beam splitter simultaneously, providing beam manipulation of the intensity distribution of the diffracted light in spherical space. We highlight here that in addition to the RPC, an artificial Lambertian scatterer featuring a constant radiance and independent of the observation angle is another promising device that can be realized using our scrambling metasurface. Our designed scrambling metasurface acts as an ideal Lambertian scatterer that scatters an incident laser beam into the entire field of view (360°) with the same omnidirectional brightness. The possible applications of an artificial Lambertian scatterer include laser lighting in the fields of consumer electronics, industry, and military defense.

Although our proposed scrambling metasurface has many potential applications, it has its own limitations. The zero-th order diffraction can be observed in our full-space spot generation experiment, which illustrates that our scrambling metasurface is still not a perfect metasurface. Future efforts in mitigating the zero-th order diffraction effect, improving the efficiency in the visible regime and equalizing the spot intensity are still necessary to enhance the performance of our scrambling metasurface.

In summary, we demonstrated a simple, flexible and extensible method for high-end beam manipulation, enabled by a high-performance, full-space, scrambling metasurface that controls the propagation direction of thousands of diffraction beams in full space. In general, our proposed beam manipulation technique overcomes the limitations of traditional DOEs that work only in transmission or reflection with limited beam manipulation areas. Furthermore, the proposed scrambling metasurface has many unparalleled advantages, such as one-step lithography, ultra-compactness and compatibility with semiconductor technology. This scrambling metasurface-based beam manipulation technique has huge potential in broad applications, such as facial recognition, 3D metrology, and 3D depth cameras.

## Methods

### Unit-cell design and simulation method

Our strategy of designing a scrambling metasurface is to find the magnetic resonance in the silicon nanobrick at the design wavelength first and then combine it with the geometric phase. After that, by carefully adjusting the geometric parameters of the nanobrick in one unit cell, we can adjust the power ratio between the reflected and refracted light, while maintaining the geometric phase. We used the commercial electromagnetic simulation software COMSOL to investigate the performance of the nanobrick-based metasurfaces. In the simulations of Fig. 1e–f, we built up a nanobrick unit cell and used periodic boundary conditions along both the *x*- and *y*-axis directions. After that, we set two perfect matched layers at both ends of the simulation domain in the *z*-axis direction. In Fig. 1g, we changed the polarization states of the incident wave from LP to CP, maintaining the same boundary conditions as Fig. 1e–f. The transmitted and reflected beams with cross-polarized and co-polarized components were collected by different field ports. Furthermore, we swept the geometric parameters of the nanobrick (length *L*, width *W*, height *H*, and cell size *C*) to optimize the performance.

### Sample fabrication

Amorphous silicon was deposited on a 500 μm thick fused silica substrate through plasma-enhanced chemical vapor deposition (BMR Technology, HiDep-SC). The nanobrick structures were patterned with a positive tone polymethyl methacrylate (PMMA) (Microchem, 495 PMMA A2) resist by standard electron beam lithography (ELIONIX, ELS-7800, 80 kV, 50 pA). The resist layer was spin-coated (2000 r.p.m., 60 s) and baked at 180 °C on a hot plate for 5 min, with a final thickness of approximately 100 nm. To prevent charging effects from the dielectric substrate, a conductive polymer (Showa Denko, E-spacer 300Z) was spin-coated (2000 r.p.m., 60 s) before the electron beam exposure step. The electron beam exposure dose was approximately 1280~1,600 μC/cm^2^. After exposure, the conductive layer was removed in deionized water and the PMMA resist was developed in methyl isobutyl ketone/isopropyl alcohol (IPA) 1:3 solutions for 12 min at 0 °C and rinsed by IPA for 30 sec. After the development process, Cr (40 nm) was deposited by electron-beam evaporation (KVT, KVE-ENS4004), followed by a lift-off process in 50 °C hot acetone. The patterned Cr layer was used as an etch mask for silicon. The silicon layer in the Cr-free part was removed using a dry etch. After the etching process, the Cr mask was removed by a Cr etchant. Finally, silicon nanobricks were formed on the substrate.

### Generation of the cloud of random points

As an RPC contains 100 × 100 pixels in one period, our design example has 100 × 100 diffraction orders for such a periodic and sub-wavelength element. However, with the operation wavelength of 633 nm and pixel size of 300 × 300 nm^2^, only 6924 diffraction sub-beams are propagating waves that can reach the far field. Here, we randomly selected 2022 sub-beams (~30%) and took them as a target pattern for phase optimization. To eliminate the polarization dependence existing in the PB phase-based elements, the matrix of the target pattern was designed with rotational symmetry around the optical axis. That is, each spot with coordinate (*x*,*y*) is equal to its conjugate spot with coordinate (–*x*, –*y*). With such an arrangement, the diffraction pattern of the LCP light will coincide with that of the RCP light exactly in the far field, and the RPC will be insensitive to the polarization state of the incident beam. More details about the RPC design are provided in the Supplementary Information.

It should be noted that since we took the spatial frequency as the propagation vector in the Fourier transformation from the surface of the RPC to the far field, the sampling in spherical space has to be non-uniform. Since the spatial frequency is defined as sin*θ*/λ, where *θ* is the diffraction angle in the *x*- or *y*-axis direction, both the angular separation and width are nonlinear. As a result, the angular separation of spots with higher orders is larger than that of spots with lower orders. In addition, the angular width of each diffraction order differs from each other and broadens again for higher orders. These problems are unavoidable in principle; however, we can use high-density spot arrays (corresponding to a larger period of the RPC) to shrink the angular separations and a larger sample (a greater number of periods) to reduce the angular width.

## Electronic supplementary material

Video

Supplementary Information
