# Supplementary material for: Full-space Cloud of Random Points with a Scrambling Metasurface
Source: Light Sci Appl. 2018 Sep 12;7:63. doi: 10.1038/s41377-018-0064-3 (PMC6134062; doi:10.1038/s41377-018-0064-3)
Supplement: Supplementary file 2 — Supplementary Information [file 41377_2018_64_MOESM2_ESM.docx]

Full-space Cloud of Random Points with a Scrambling Metasurface: **Supplementary Information**

Zile Li^1,2#^, Qi Dai^1,2#^, Muhammad Q. Mehmood^3,4#^, Guangwei Hu^4,5#^, Boris Luk’yanchuk^6^, Jin Tao^2^, Chenglong Hao^4^, Inki Kim^7^, Heonyeong Jeong^7^, Guoxing Zheng^1,2*^, Shaohua Yu^2*^, Andrea Alu^5^, Junsuk Rho^7,8,9*^, and Cheng-Wei Qiu^4*^

*^1^School of Electronic Information, Wuhan University, Wuhan 430072, China*

*^2^NOEIC, State Key Laboratory of Optical Communication Technologies and Networks, Wuhan Research Institute of Posts & Telecommunications, Wuhan 430074, China*

*^3^Department of Electrical Engineering, Information Technology University of the Punjab, Ferozpur Road, 54000 Lahore, Pakistan*

*^4^Department of Electrical and Computer Engineering, National University of Singapore, 4 Engineering Drive 3, Singapore, 117583, Singapore*

*^5^Advanced Science Research Center, City University of New York, New York 10031, USA*

*^6^Data Storage Institute, A*STAR (Agency for Science, Technology and Research), 2 Fusionopolis Way, #08-01, Innovis 138634, Singapore*

*^7^Department of Mechanical Engineering, Pohang University of Science and Technology (POSTECH), Pohang 37673, Republic of Korea*

*^8^Department of Chemical Engineering, Pohang University of Science and Technology (POSTECH), Pohang 37673, Republic of Korea*

*^9^National Institute of Nanomaterials Technology (NINT), Pohang 37673, Republic of Korea*

**1. The measured refractive index of amorphous silicon.**

The measured refractive index of the amorphous silicon used in our work versus wavelength is plotted in Fig. S1. At the design wavelength 630 nm, the real part is 3.249 and the imaginary part is 0.047.


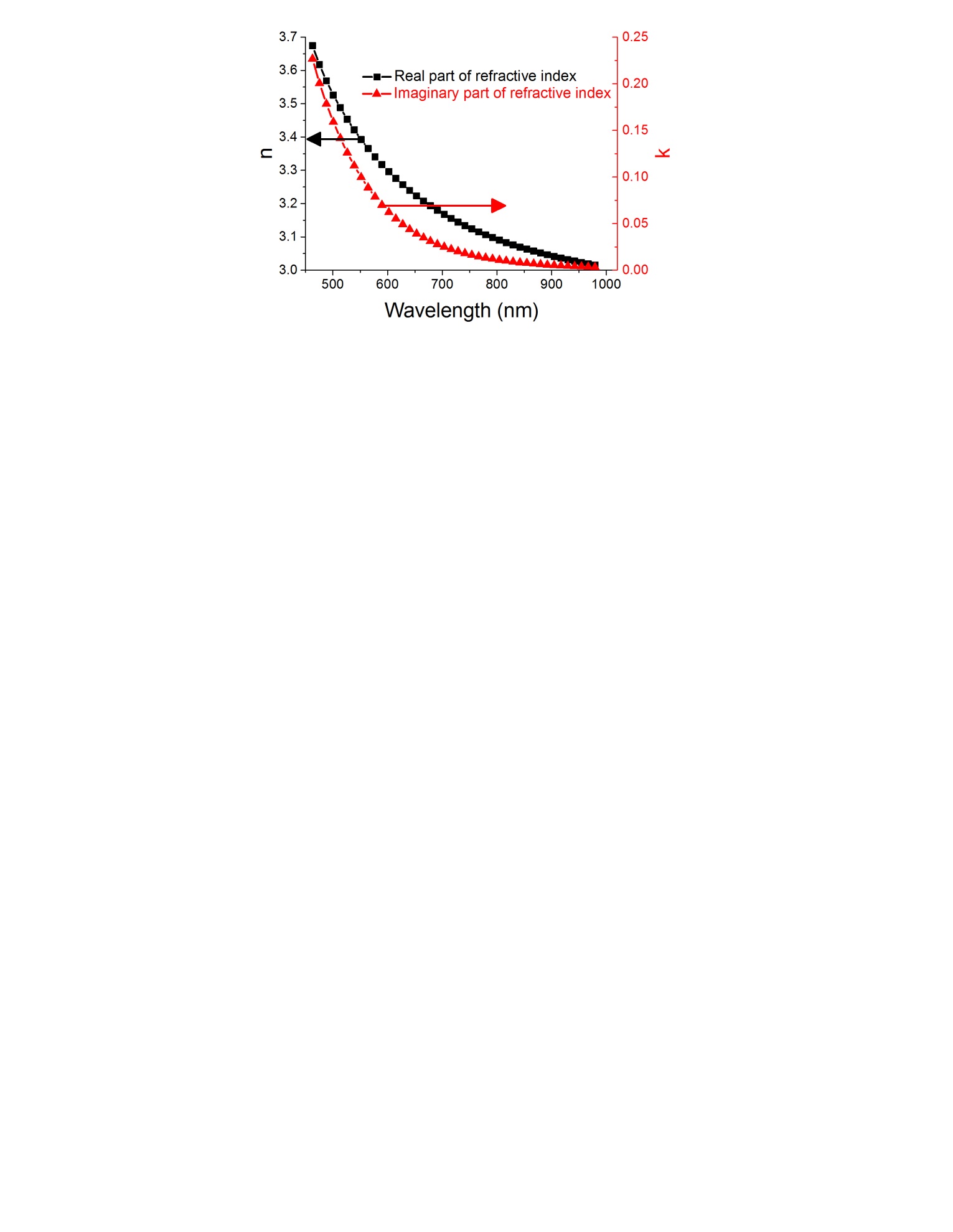


**Supplementary Fig. S1:** The measured refractive index of amorphous silicon.

**2. Design and simulation of a scrambling metasurface operating in infrared ranges.**

In the main text, the scrambling metasurface was designed at a wavelength of 630 nm for observation convenience. However, the high loss at visible light existing in amorphous silicon leads to a relatively low efficiency (~27% for both reflection and transmission). In this chapter, we demonstrate that, scrambling metasurface can be designed with efficiency as high as 85% totally (reflection and transmission together) in infrared ranges (810-830 nm), shown in Fig. S2.


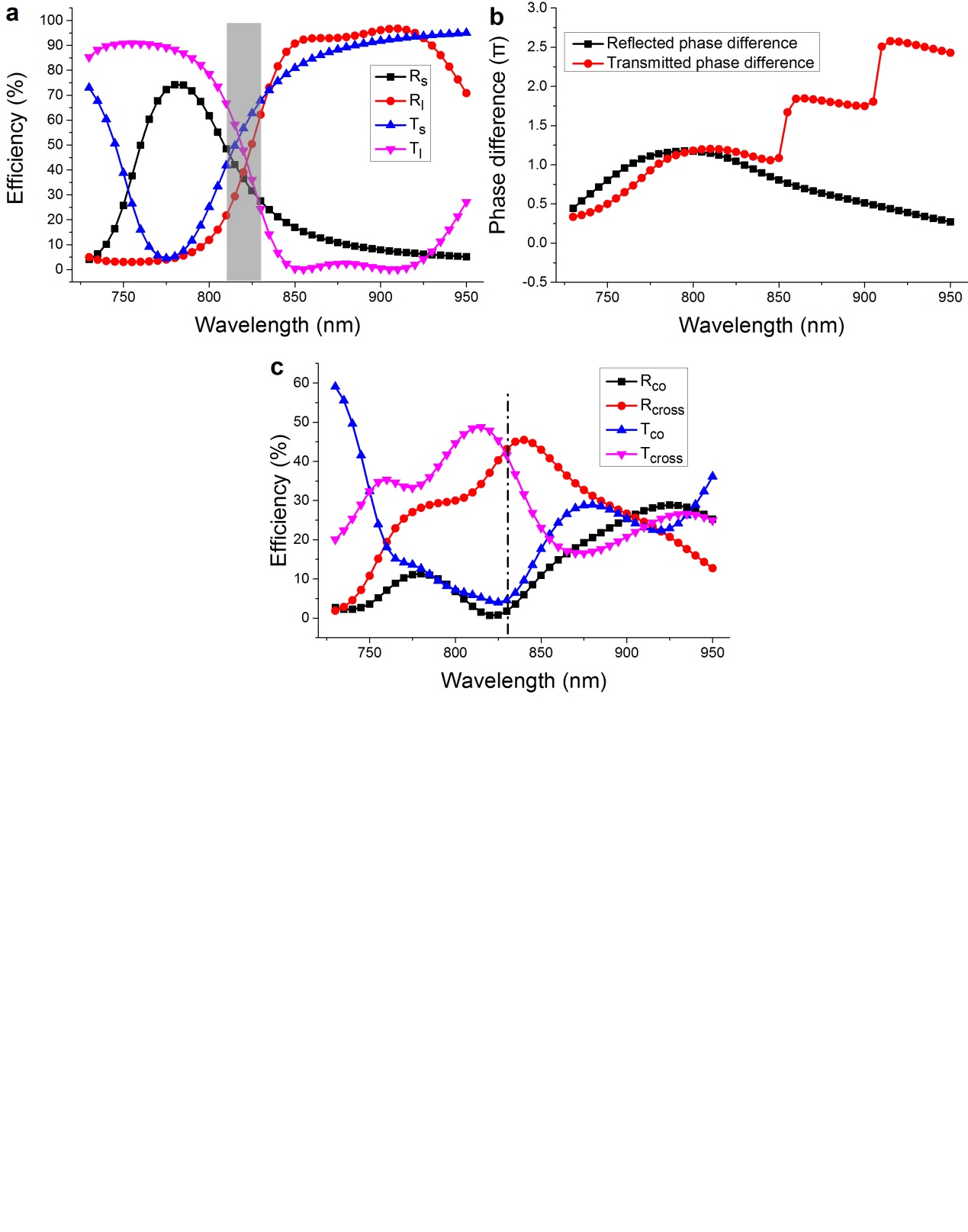


**Supplementary Fig. S2:** Illustration of the unit-cell by numerical simulations. **a,** Reflectivity and transmissivity versus wavelength when an incident beam is linearly polarized along the long and short axes of the nanobrick, respectively. The amorphous silicon nanobrick cell was designed with height H of 310 nm, length L of 200 nm, width W of 120 nm, and cell size C of 400 nm. The reflectivity and transmissivity are labeled as *R_s_*, *T_s_*, *R_l_* and *T_l_* when waves propagate with polarization direction along the short and long axes of the nanobricks, respectively. The grey bar illustrates the wavelength range between 810-830 nm where the reflectivity and transmissivity are approximately equal. **b,** The phase differences between the long and short axes for both reflection and transmission. **c,** Simulated efficiency of reflected co-polarized part (R_co_) and cross-polarized part (R_cross_), transmitted co-polarized part (T_co_) and cross-polarized part (T_cross_) versus wavelength.

**3. Design and simulation of a scrambling metasurface with specified power ratio.**

As examples, aside from the demonstrated scrambling metasurface with the equal transmissivity and reflectivity mentioned above and in main text, we also designed three other nanobrick phase modulators working with: totally transmission; totally reflection; and imbalanced transmissivity and reflectivity (the ratio between transmission and reflection is 3: 1). We simulated the efficiency of co-polarized and cross-polarized parts for both reflection and transmission of the output beam versus wavelength, shown in Fig. S3. For the optimized geometry configuration upon normal light incidence, the numerical simulations show that for both reflection and transmission the cross-polarized sub-beams go exactly into the expected parts; meanwhile, the unwanted co-polarized parts are extremely low (less than 5%), which proves the effectiveness of the proposed metasurfaces to control both the propagation direction and the phase of an incident beam.


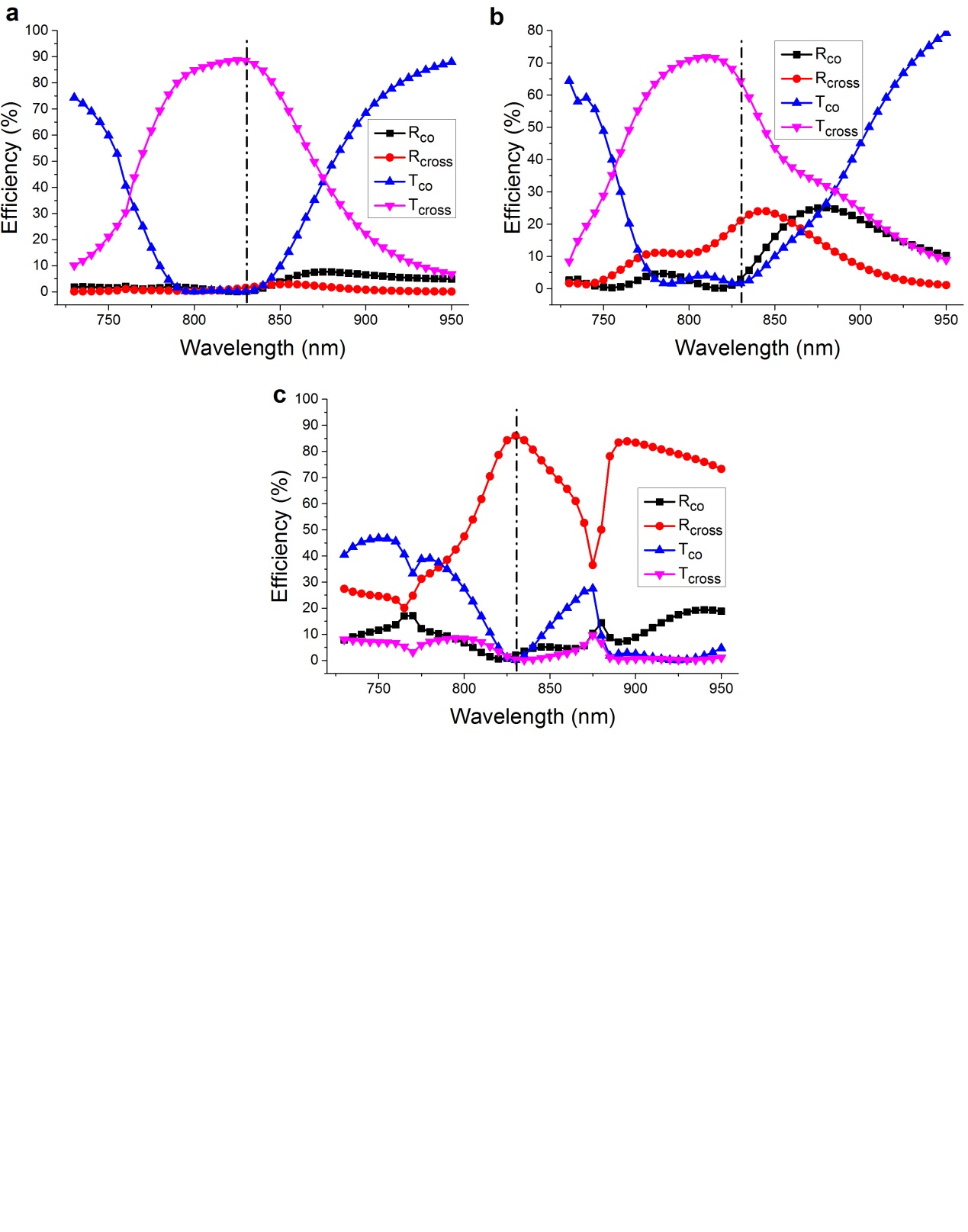
**Supplementary Fig. S3:** Simulated efficiency of reflected co-polarized part (R_co_) and cross-polarized part (R_cross_), transmitted co-polarized part (T_co_) and cross-polarized part (T_cross_) versus wavelengths. **a,** The silicon nanobrick cell was designed with height H of 420 nm, length L of 160 nm, width W of 120 nm, and cell size C of 400 nm. This kind of nanobrick can act as a totally transmitted phase modulator. **b,** The silicon nanobrick cell was designed with height H of 360 nm, length L of 170 nm, width W of 120 nm, and cell size C of 400 nm. This kind of nanobrick can act as an unequal phase modulator (3:1 between transmitted and reflected light). **c,** The silicon nanobrick cell was designed with height H of 280 nm, length L of 340 nm, width W of 150 nm, and cell size C of 450 nm. This kind of nanobrick can act as a totally reflected phase modulator. The designed wavelength 830 nm has been denoted with dash dots in Figs. (a-c).

Finally, we designed a dual-focus metalens to verify the effectiveness of above scrambling metasurface. To achieve the phase profile equivalent to a conventional cylindrical lens, the relationship between the orientation angle *φ*(*x*) and the location *x* of nanobrick is governed by

$2\phi(r)=\pm\frac{2\pi}{\lambda}(\sqrt{r^{2}+{f'}^{2}}-\left| f' \right|)$, (1)

where *λ* is the free-space wavelength, *f* ' is the focal length of the metalens, and *x* is the distance of each nanobrick from the center of the metalens. Fig. S4a shows the simulated distribution of electric field magnitude |*E*|. The designed focal length is 3 μm and the incident beam is a circularly-polarized plane-wave with a wavelength of 830 nm. It is shown that beam focusing appears both in object and image spaces with almost equal intensity and the phase distribution shown in Fig. S4b denotes two focal planes (the location is around the centers of two converging spherical waves at 3 μm). We can also change the energy ratio of the two focuses by simply changing the dimension of each nanobrick (the structural parameters have been listed in the caption of Fig. S2) and the beam focusing effects are shown in Fig. S4c-e. To observe the positions of beam focusing, we plot the intensity |*E*|^2^ along the optical axis in Fig. S4f. It is shown that all of the simulated results agree well with our design.


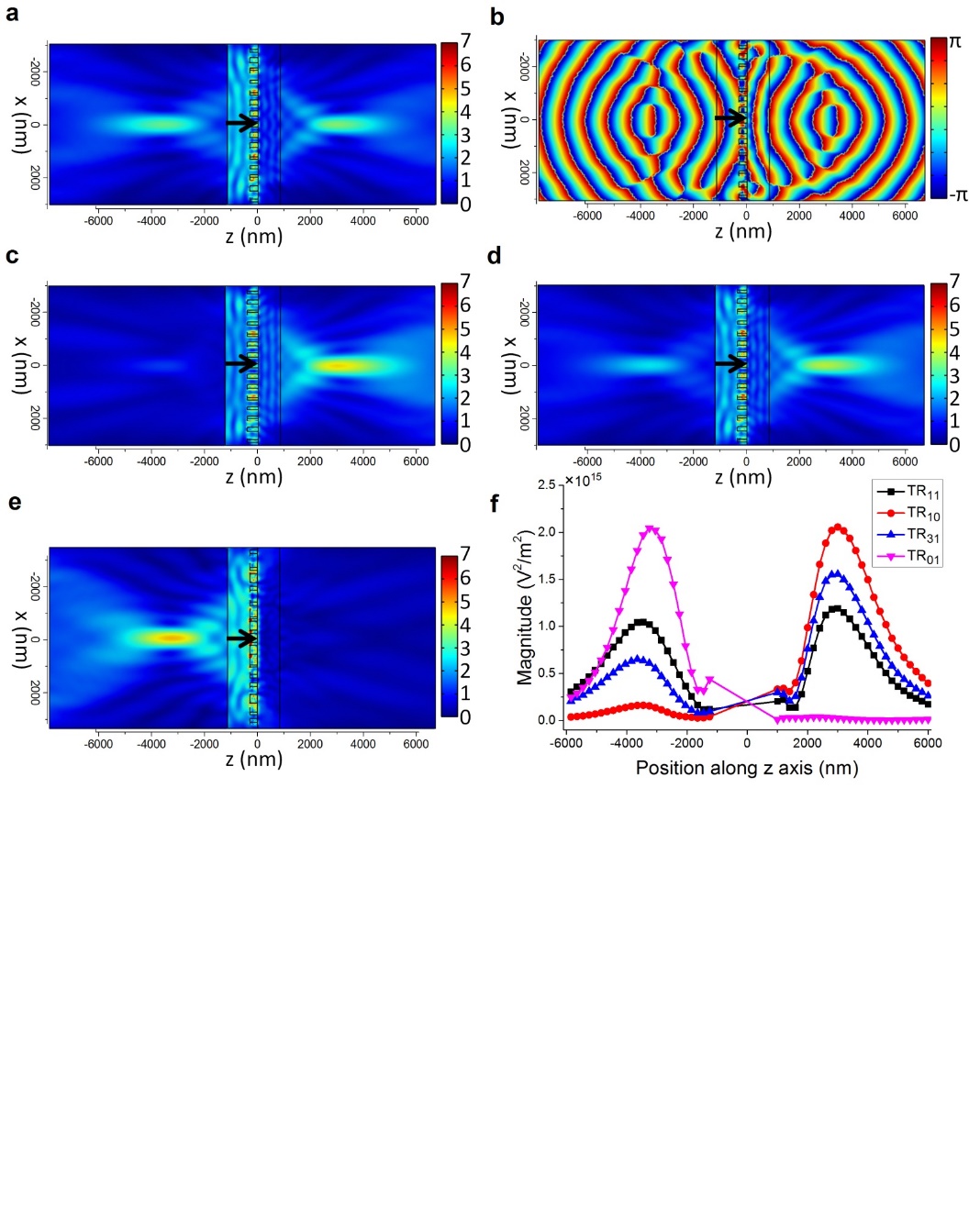


**Supplementary Fig. 4: a** **and** **b,** Intensity and phase distribution of electric field in the simulation domain with a CP light incidence upon a dual-focus metalens (equal transmission and reflection). **c-e,** The simulated intensity distribution of a dual-focus metalens consists of totally transmitted, 3:1 splitting ratio and totally reflected phase modulator. The bold dark arrows indicate the port and propagation direction of the incident beam. **f,** Squared electric-field magnitude |E|^2^ (intensity) along the optical axis of the four metalenses in **a** and **c-e**, which are marked as TR_11_, TR_10_, TR_31_, and TR_01_, respectively. Considering that we care only the positions of beam focusing, we do not cover the data between the incident port and substrate.

**4. Design and Experimental results for a scrambling metasurface based blazed grating with different wavelengths.**

The scrambling metasurface can form a phase grating capable of directing a CP incident beam into transmitted +*m*^th^ order and equal energy into reflected +*m*^th^ order. According to the grating equation, the diffraction angle of a blazed grating equals sin^-1^[*mλ*/(*n*C)], where *n* is the cell number in one period and C is the cell size. As a design example, we chose *n*=13 and the maximum diffraction order *m*=6, corresponding a maximum diffraction angle of 75.75° (*λ*=630 nm). Since an ideal blazed grating can transform all of the diffracted light into the +*m*^th^ (or -*m*^th^) order, the phase of the GEMS grating should be quantified in 0~12π (i.e. *m*×2π), corresponding to a phase step of 12π/*n*=12π/13. Since a linearly polarized (LP) beam can be considered as a combination of a left-handedness circularly polarized (LCP) and right-handedness circularly polarized (RCP) beams with equal intensity, such transflective grating can diffract an incident beam into four sub-beams with symmetrical propagation directions and equal intensity.

The experimental result for above transflective blazed grating is shown in Fig. S5. At the operation wavelength of 630 nm, the measured diffraction angle of the 6^th^ order is about 75°, which agrees well with the theoretical calculation (75.75°). To further investigate the spectral response of the transflective blazed grating, we used a super continuum light source (YSL SC-pro) in the range from 470 to 670 nm in steps of 10 nm to illuminate the grating sample and then collected the diffraction beam in a circular truncated cone, the results are shown in Supplementary Movies and Fig. S6. The diffraction angle in designed order (*m*=6) increases with operation wavelength varies from 470 nm to 650 nm and then the 6^th^ order disappears after 650 nm since the diffraction angle exceeds 90°. We also increase one wavelength one by one until a “white” laser beam containing 8 wavelengths illuminates the sample, as shown in Fig. S7. All observed phenomena agree well with theoretical analysis, which proves the effectiveness of the proposed metasurfaces.


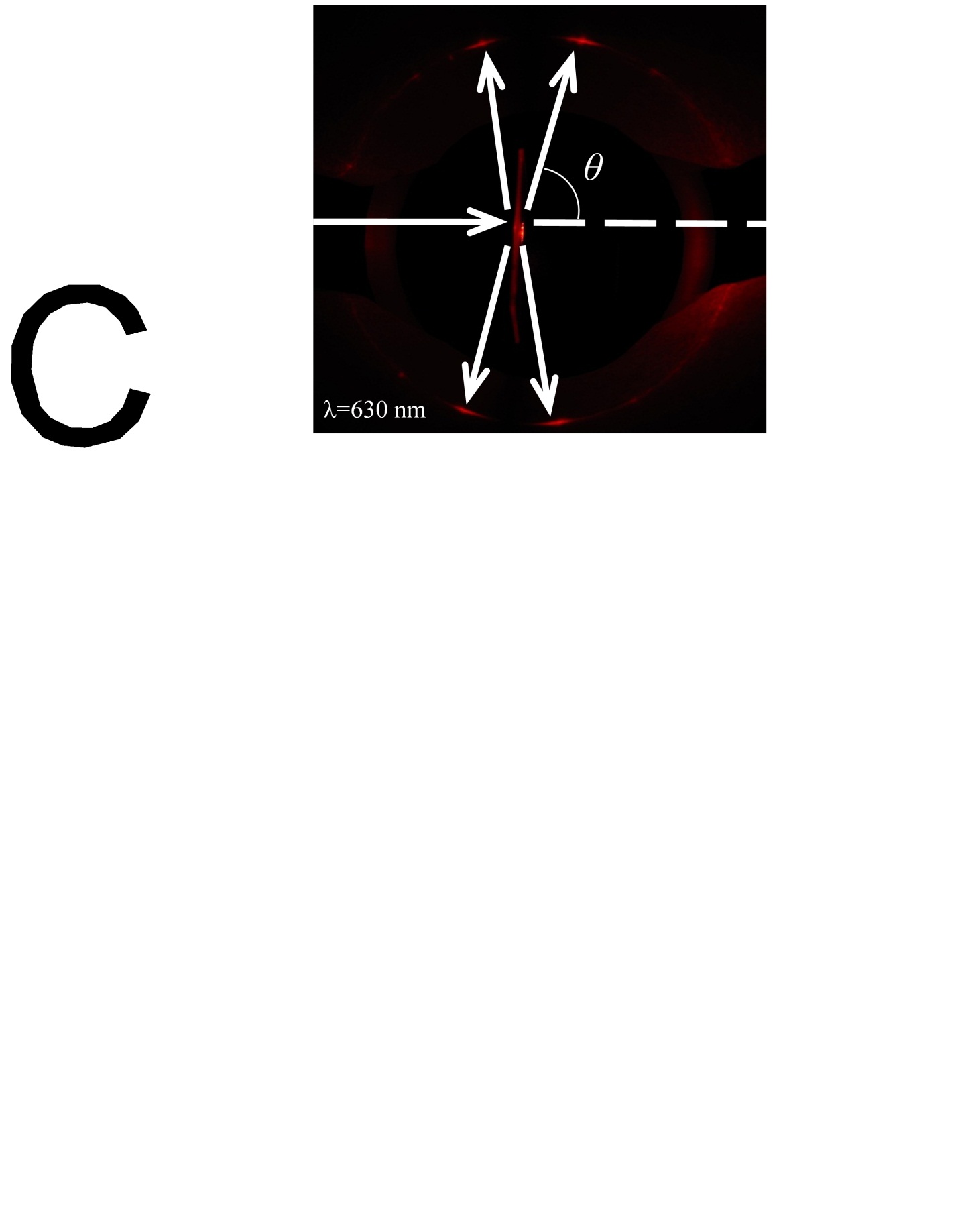


**Supplementary Fig. S5:** Experimental result of the transflective blazed grating illuminated by a supercontinuum laser, the incident wavelength is 630 nm. The white arrows indicate the directions of incident and diffracted light.


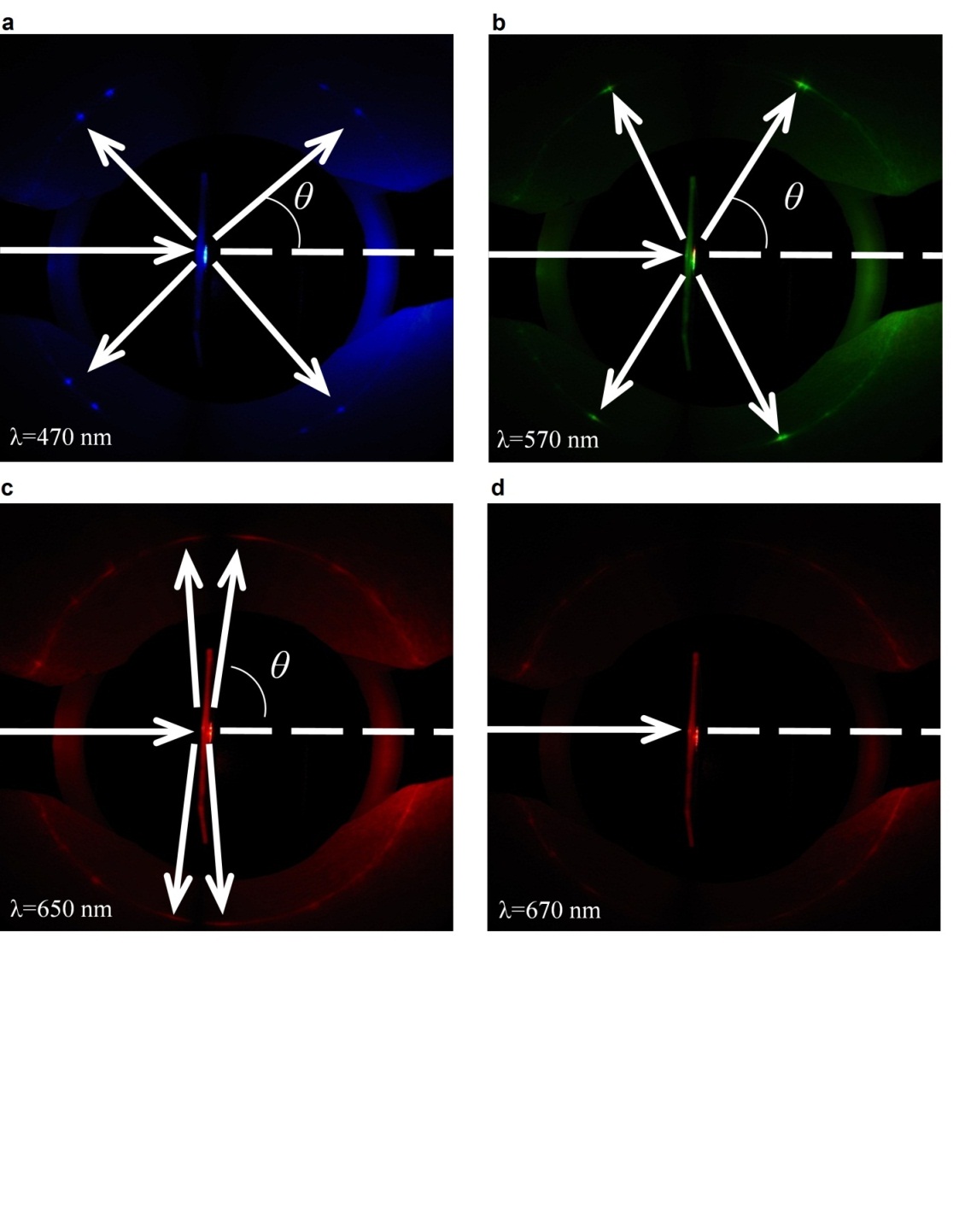


**Supplementary Fig. S6: a-d,** Experimental results of the transflective blazed grating illuminated by a supercontinuum laser, the incident wavelength is 470 nm, 570 nm, 650 nm and 670 nm, respectively. The white arrows indicate the directions of incident and diffracted light.

**
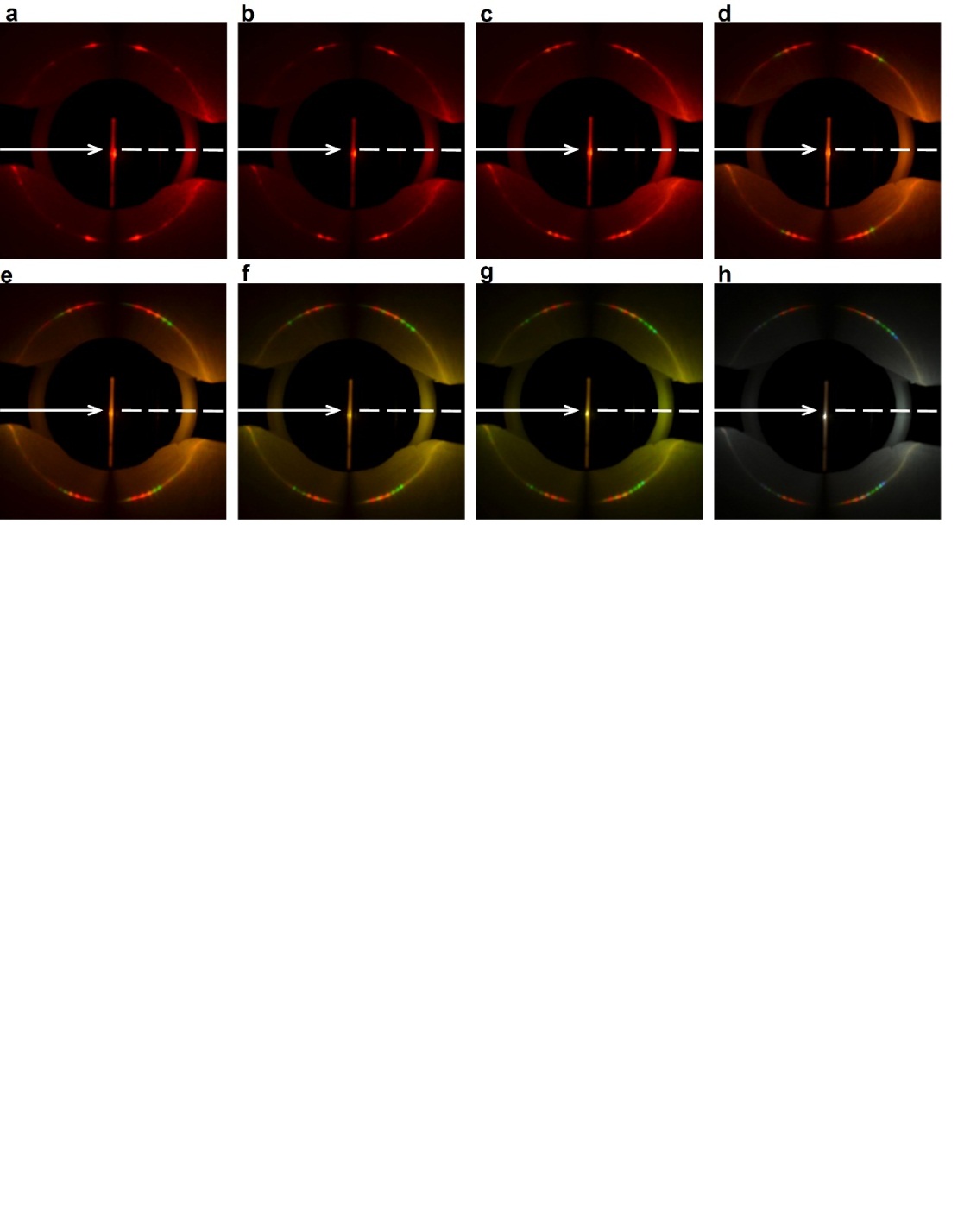
**

**Supplementary Fig. S7: a-h,** Experimental results for a scrambling metasurface based blazed grating with different wavelengths. From (a) to (h), we increase one wavelength one by one until a “white” laser beam containing 8 wavelengths (490, 510, 530, 550, 570, 590, 610 and 630 nm) illuminates the sample shown in (h).

**5. Experimental results for a scrambling metasurface based 2×2 beam splitter with different wavelengths.**


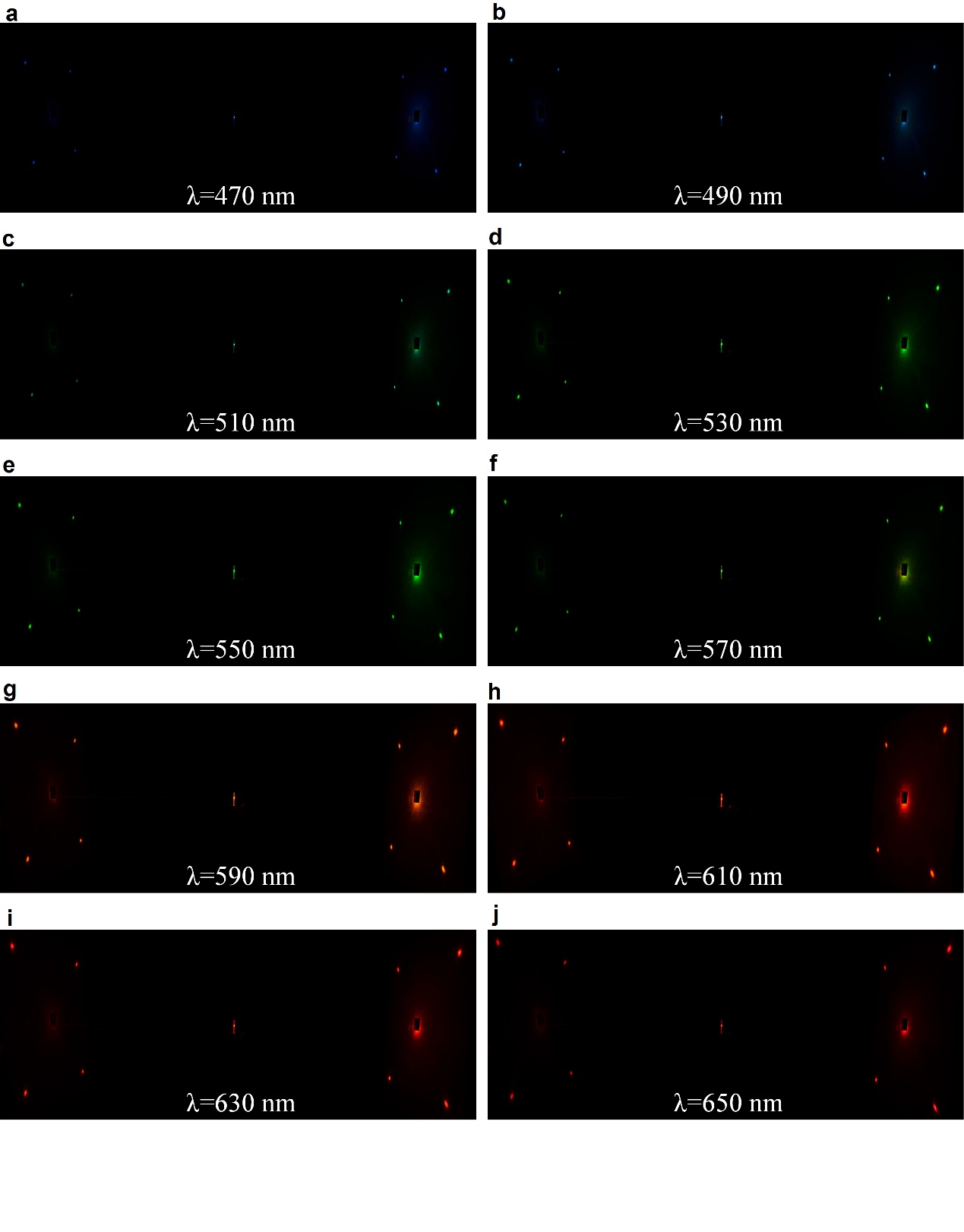


**Supplementary Fig. S8: a-j,** Experimental results for a scrambling metasurface based 2×2 beam splitter with ten different wavelengths. The operation wavelength is denoted under each figs.

**6. Design of a random point cloud (RPC) metasurface.**

For a diffractive optical element such as a RPC, we assume each period contains *M_x_* × *M_y_* unit cells, and the interval between adjacent cell centers is *C*. With a normally incident light, the numbers of diffraction orders generated in the transmission and reflection spaces are the same as the pixel numbers, that is, *M_x_* × *M_y_*. Specially, the transverse spatial frequency of the (*m_x_*, *m_y_*)th diffraction order is:

$\begin{matrix} k_{x}=m_{x}/M_{x}C,k_{y}=m_{y}/M_{y}C \\ k'=\sqrt{k_{x}^{2}+k_{y}^{2}} \end{matrix}$, (2)

where |*m_x_*|≤*M_x_*/2, |*m_y_*|≤*M_y_*/2. When the transverse spatial frequency *k*'≥1/*λ*, the corresponding diffraction order becomes evanescent wave and it cannot propagate to the far field. In contrast, when the transverse spatial frequency *k*' is less than 1/*λ*, the diffraction order corresponds to a propagation wave and it can propagate to the far field. The diffraction angle of a diffraction order is determined by:

$\theta_{x}=\arcsin(k_{x}\lambda),\theta_{y}=\arcsin(k_{y}\lambda),\theta=\arcsin(k'\lambda)$, (3)

where *θ_x_* is the angle between the diffraction order and the coordinate plane *yoz*, *θ_y_* is the angle between the diffraction order and the coordinate plane *xoz*, and *θ* is the angle between the diffraction order and the *z*-axis. From equations (2) and (3), one can see that when the center interval between the unit cells satisfies *C*≤*λ*/2, the diffraction angle of a propagation wave can approach 90°, i.e., the diffracted sub-beams of a scrambling metasurface can fill a full space. We selected the diffraction orders with a number of *N_b_* randomly in the propagation waves and took them as the target diffraction orders. The program chart of designing the target diffraction orders is shown in Fig. S9. It should be noted that, to avoid the patterns overlapping with each other for LCP and RCP incident light, the target diffraction orders were designed to satisfy the rotational symmetry around the optical axis. That is, the (*m_x_*, *m_y_*)th order has the same intensity as the (-*m_x_*, -*m_y_*)th order.


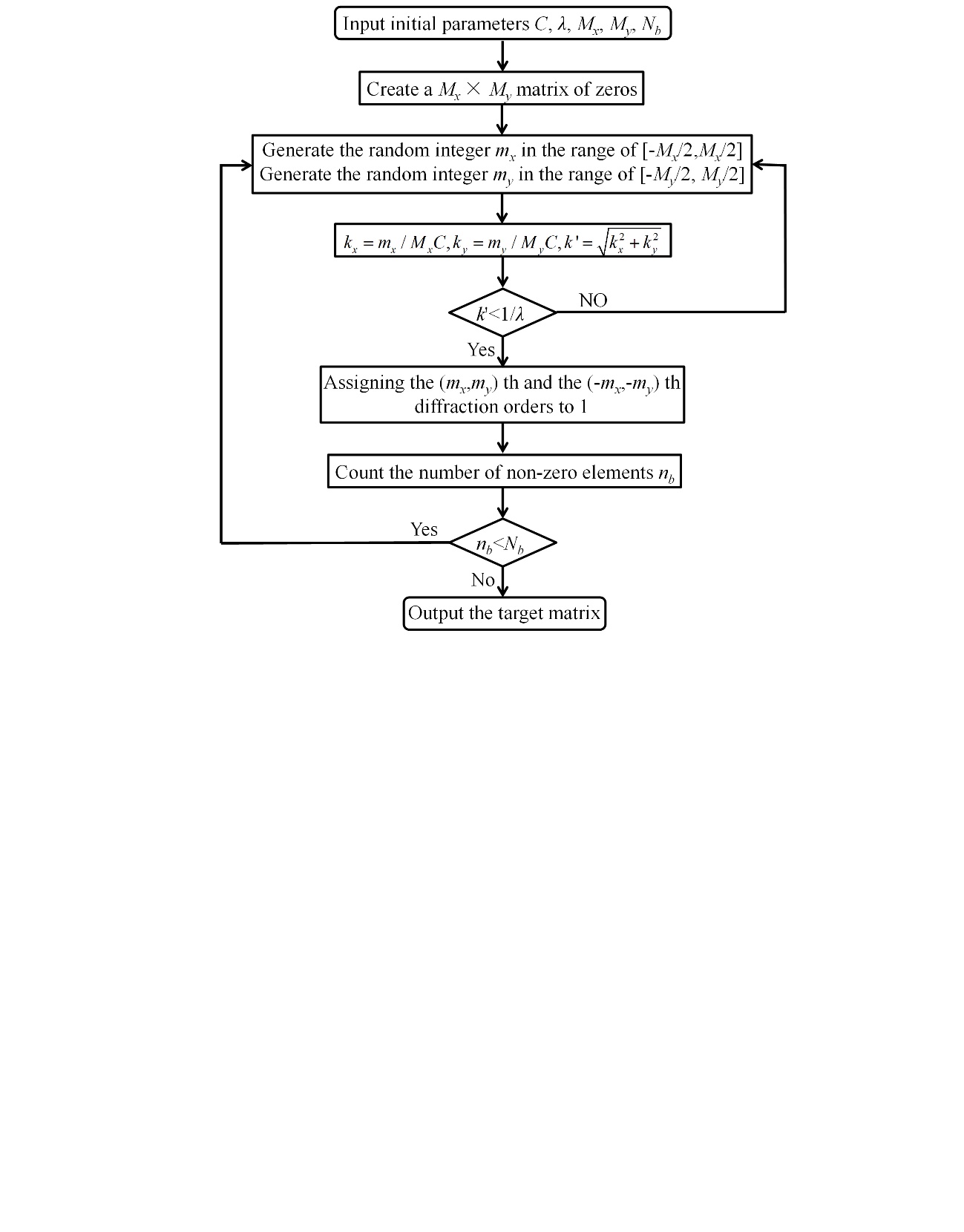


**Supplementary Fig. S9:** The design program chart to generate target diffraction orders.

The designed RPC has a dimension of 300×300 μm^2^ and contains 10×10 periods. The size of each period is 30×30 μm^2^ and the number of nano-bricks is 100×100.When the wavelength of the incident beam is *λ*=632.8 nm, it was calculated that there are 6,924 diffraction orders being propagation waves in both the transmission and reflection space. Because each unit cell has a dimension of *C*=300 nm and satisfies *C*≤*λ*/2, the diffraction light wave can fill a full space. As a design example, we selected *N_b_*=2,022 and the target spots (diffraction orders) is shown in Fig. S10.


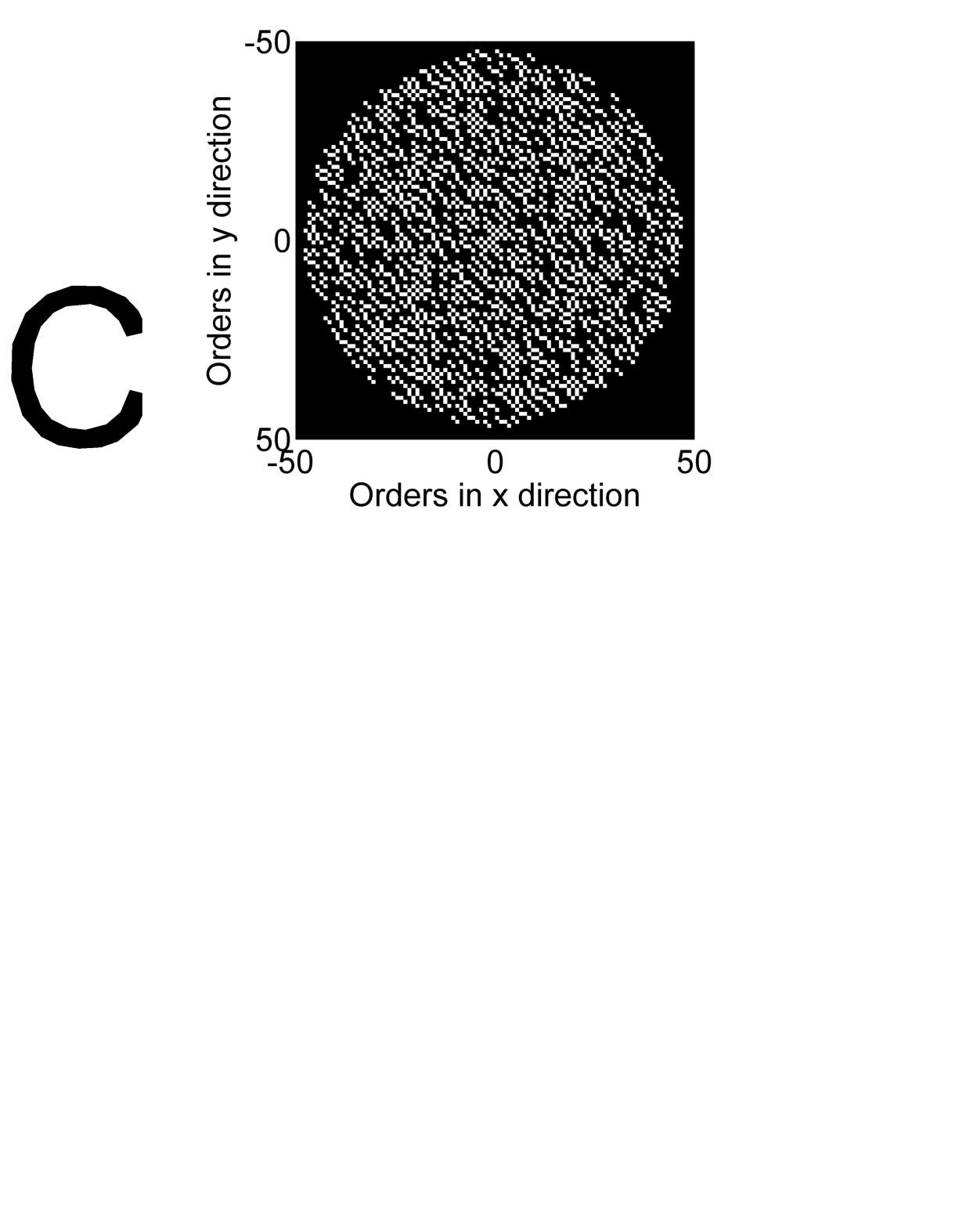


**Supplementary Fig. S10:** Target spots in the transmission or reflection spaces.

To generate the random spot arrays illustrated in Fig. S10, the phase distribution of the RPC is optimized by the classic Gerchberg-Saxton algorithm. After that, the orientation angle of each nanobrick in the RPC is determined since the geometric phase is exactly two times of the orientation angle.
